# Agile Acceptance Test–Driven Development of Clinical Decision Support Advisories: Feasibility of Using Open Source Software

**DOI:** 10.2196/medinform.9679

**Published:** 2018-04-13

**Authors:** Mujeeb A Basit, Krystal L Baldwin, Vaishnavi Kannan, Emily L Flahaven, Cassandra J Parks, Jason M Ott, Duwayne L Willett

**Affiliations:** ^1^ University of Texas Southwestern Medical Center Dallas, TX United States

**Keywords:** clinical decision support systems, electronic health records, software validation, software verification, agile methods, test driven development

## Abstract

**Background:**

Moving to electronic health records (EHRs) confers substantial benefits but risks unintended consequences. Modern EHRs consist of complex software code with extensive local configurability options, which can introduce defects. Defects in clinical decision support (CDS) tools are surprisingly common. Feasible approaches to prevent and detect defects in EHR configuration, including CDS tools, are needed. In complex software systems, use of test–driven development and automated regression testing promotes reliability. Test–driven development encourages modular, testable design and expanding regression test coverage. Automated regression test suites improve software quality, providing a “safety net” for future software modifications. Each automated acceptance test serves multiple purposes, as requirements (prior to build), acceptance testing (on completion of build), regression testing (once live), and “living” design documentation. Rapid-cycle development or “agile” methods are being successfully applied to CDS development. The agile practice of automated test–driven development is not widely adopted, perhaps because most EHR software code is vendor-developed. However, key CDS advisory configuration design decisions and rules stored in the EHR may prove amenable to automated testing as “executable requirements.”

**Objective:**

We aimed to establish feasibility of acceptance test–driven development of clinical decision support advisories in a commonly used EHR, using an open source automated acceptance testing framework (FitNesse).

**Methods:**

Acceptance tests were initially constructed as spreadsheet tables to facilitate clinical review. Each table specified one aspect of the CDS advisory’s expected behavior. Table contents were then imported into a test suite in FitNesse, which queried the EHR database to automate testing. Tests and corresponding CDS configuration were migrated together from the development environment to production, with tests becoming part of the production regression test suite.

**Results:**

We used test–driven development to construct a new CDS tool advising Emergency Department nurses to perform a swallowing assessment prior to administering oral medication to a patient with suspected stroke. Test tables specified desired behavior for (1) applicable clinical settings, (2) triggering action, (3) rule logic, (4) user interface, and (5) system actions in response to user input. Automated test suite results for the “executable requirements” are shown prior to building the CDS alert, during build, and after successful build.

**Conclusions:**

Automated acceptance test–driven development and continuous regression testing of CDS configuration in a commercial EHR proves feasible with open source software. Automated test–driven development offers one potential contribution to achieving high-reliability EHR configuration. Vetting acceptance tests with clinicians elicits their input on crucial configuration details early during initial CDS design and iteratively during rapid-cycle optimization.

## Introduction

### Defects in Clinical Decision Support Tools

“Making the right thing the easy thing to do” for clinicians using an electronic health record (EHR) drives many current efforts to promote delivery of reliable, high-quality care. Clinical decision support (CDS) within the EHR provides one mechanism, by supplying advisories suggesting best practice care for a patient’s specific conditions [1,2].

With the move to EHRs came recognition that unintended consequences can ensue [3,4], even jeopardizing patient safety [5,6]. Modern EHRs comprise complex software code with extensive local configurability options, affording opportunities for defects to be introduced. Feasible approaches to prevent and detect defects in EHR configuration are needed.

Defects in CDS tools are surprisingly common and can cause either over-expression or under-expression of alerts [7-9]. The latter can go undetected for long periods. Common causes of CDS defects include changes to data codes, terminologies, or modules external to the CDS itself [7].

### Test–Driven Development

**Figure 1:**
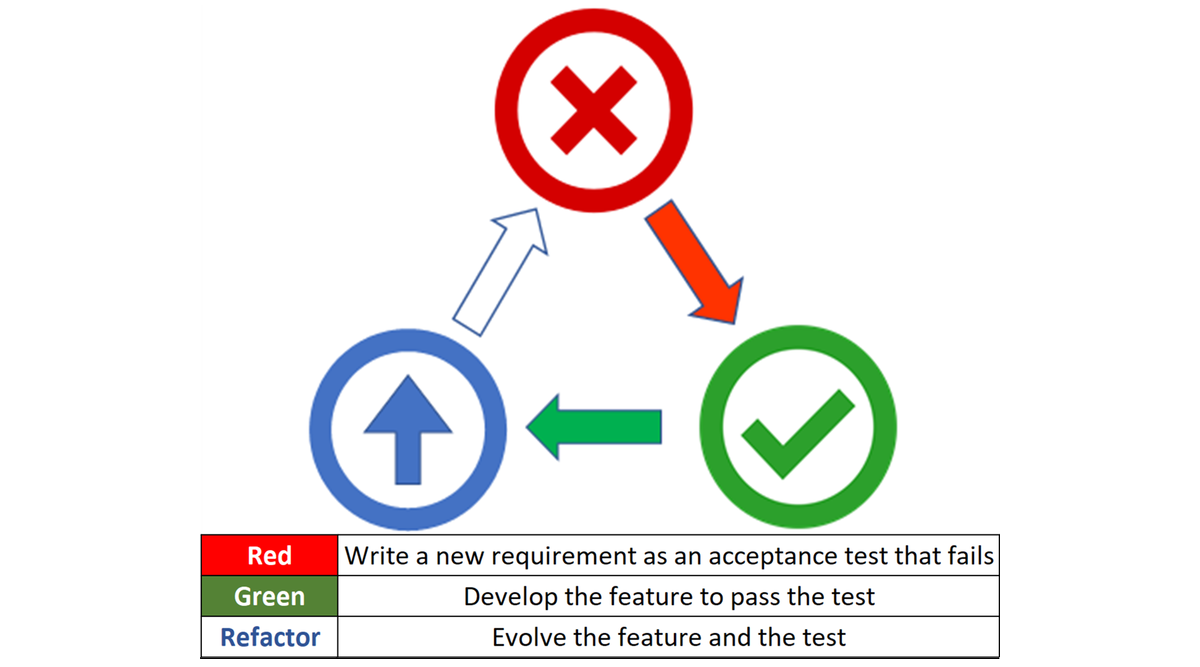
Test–driven development cycle.

In complex software systems, use of test–driven development (TDD) and automated regression testing promotes reliability [10,11]. In TDD, a new requirement is specified as a test before code is written, following a “red-green-refactor” pattern: the test fails initially (“red”) then passes once the software meets all test-specified requirements (“green”). Subsequent refinements to the underlying code (refactoring) can occur, following the same cycle (Figure 1).

Benefits of TDD include (1) encouragement of modular design and (2) growth of regression test suites. Automated regression test suites improve software quality and provide a “safety net” for later modification without fear of undetected breakage [12]. TDD can be done at the micro (unit test) and macro (acceptance test) levels. Each automated acceptance test serves multiple purposes, as requirements definition (prior to build), acceptance testing (on completion of build), regression testing (after go-live), and documentation of design (for long-term reference) [13].

### Potential Applications of Acceptance Test–Driven Development for Clinical Decision Support Advisories

Rapid-cycle development or “agile” methods are being successfully applied to CDS development [14-16]. The agile practice of automated TDD is not widely adopted, perhaps because most EHR software is vendor-developed. However, key CDS advisory configuration design decisions amenable to automated testing as “executable requirements” include [17]:

- any *restrictions* on where the CDS alert logic should be evaluated (ie, restricted to only certain practice locations, encounter types, provider types) to help target the most appropriate situations and limit “alert fatigue” [18,19]
- *triggering action(s)* that prompt evaluation of the CDS advisory logic at the right time in the workflow (eg, opening the chart, placing an order, entering a diagnosis, and other options)
- *rule logic* for evaluating whether the advisory should display (“fire”), decided by evaluating discrete data in the EHR
- the *user interface* (UI) displayed after the rule logic passes, including instructions and contextual information presented, and the range of action options provided
- *system actions and state changes* that should occur in the EHR following any clinician interactions with the UI.

In this paper, we use examples of each of the above (in a widely used EHR) to demonstrate how TDD of CDS advisories can work in practice during development of a CDS tool.

### Clinical Background for an Example Clinical Decision Support Request

Patients who present to the emergency room with an acute stroke may have impaired swallowing mechanisms. Attempting to give medications orally creates a risk of the patient aspirating medication into the lungs. Accordingly, patients with known or suspected stroke are screened for swallowing difficulties prior to attempting administration of oral medication. In a busy emergency room setting, keeping track of whether the needed screening has been done can be challenging. Accordingly, interruptive CDS was requested if the intended swallow screening had not yet occurred.

## Methods

### Location

All activities in this report took place at the University of Texas Southwestern Medical Center in Dallas, Texas. This work was judged not to be human subjects research and thus did not require presentation to our Institutional Review Board.

### Software

Automated testing employed the open source testing software FitNesse, based on the Framework for Integrated Testing, along with the dbFit extension for querying databases [20-22]. Time and personnel requirement estimates for initial configuration of FitNesse and dbFit testing framework are given in Table 1. Electronic health record software at UT Southwestern is from Epic, and the incident management software is ServiceNow.

### Procedures

#### High-Level Requirements With User Stories

Initial high-level requirements for new CDS advisories were gathered as user stories [23], written from the perspective of the clinician receiving the alert: “As a <clinician role>, I want <to be advised about something>, so that <a benefit can be achieved>”.

Through clinical conversations, user stories were elaborated with more specific acceptance criteria describing what would constitute successful CDS advisory behavior, often initially as a simple bulleted list. In this project, certain acceptance criteria were further detailed unambiguously as automatable acceptance tests.

#### Automated Acceptance Test–Driven Development

Acceptance tests were initially constructed as tables in a spreadsheet (Microsoft Excel workbook), to facilitate clinical vetting and shared review. Each table specified one configuration aspect of the CDS advisory. Table contents were then imported into an automated test suite in FitNesse (see the earlier section, Software). For each table-based test, a structured query language (SQL) query retrieved the corresponding CDS advisory configuration information from the EHR development environment’s database. A FitNesse test suite template was created containing the most frequently used specification tables and corresponding SQL queries, streamlining test generation for each new CDS advisory.

For configuration management, both the CDS advisory and its associated test were migrated from the development environment to the test environment at the same time, for integrated testing. Similarly, when migrating to production, the corresponding FitNesse test(s) were added to the automated regression test suite for the production environment.

**Table 1:** Configuration of FitNesse and dbFit: time and personnel requirements. EHR: electronic health record; IT: information technology; SQL: structured query language.

Task category	Task	Frequency	Time (range)	Type of personnel
Initial set-up of FitNesse + dbFit testing framework	Download and install FitNesse to point of functioning FitNesse wiki	Once	30 minutes	IT analyst
	Configure FitNesse to use Active Directory login permissions (if desired)	Once	2 hours to 1 day	IT analyst knowledgeable about one’s local Active Directory
	Configure dbFit	Once	Few minutes to 2 hours	IT analyst
	Set up database connection for FitNesse/dbFit to query an EHR (or other) database	Once per database	1 hour (if first time doing); a few minutes per connection once experienced	IT analyst
Create a test “template” for a given type of test	Write SQL to serve as template for given type of test	Once per new type of test	1 to 2 hours	EHR analyst; SQL writer (can be same person)
Configure an individual test instance	Create Microsoft Excel copy of test template and populate for given test instance, ready for vetting with clinician or other customer	Once per test instance	15 to 60 minutes	EHR analyst
	Import Microsoft Excel test to FitNesse Test page, and test	Once per test instance	10 to 15 minutes	EHR analyst or test team analyst

Any subsequent failures of regression tests in production would initiate a new entry in the incident management system for investigation and resolution.

#### Requirements Elicitation

A nurse informaticist (EF) and an EHR analyst (JO) met with the front-line nurses and nurse manager from the Emergency Department (ED) to define the problem and frame the user story for the alert in a way these nurse clinicians believed would be beneficial within their workflow. The same EHR analyst also had standing meetings with the ED nursing and medical staff at least weekly; those sessions were used to further elaborate more detailed acceptance criteria for the user story.

## Results

### User Story for a Clinical Decision Support Best Practice Advisory

“As an emergency room nurse, I want to be alerted before I administer an oral medication to a patient with known or suspected stroke if they’ve not yet had their Swallow Screen performed, so that my patient can receive their medications by the most safe and effective route.”

### Automated Acceptance Tests for the Clinical Decision Support Advisory

#### Restrictions

**Figure 2:**
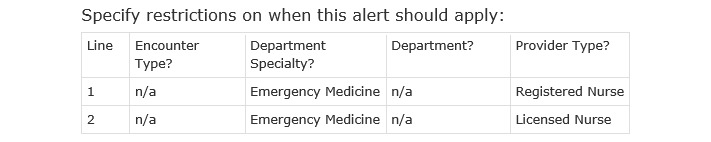
Screenshot of FitNesse test specifying Department Specialty and Provider Type restrictions. n/a: not applicable.

Restrictions help focus the advisory to the right practice setting and clinician type, reducing alert fatigue for clinicians where the advisory would not be relevant (Figure 2). This test specified that this alert should apply only in Emergency Medicine departments and only to nurses.

#### Triggering Action

**Figure 3:**
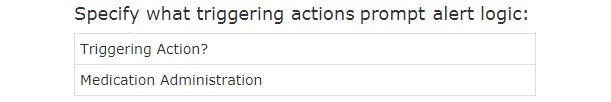
Screenshot of test specifying triggering action for this advisory.

Triggering actions further focus when the advisory’s logic should be evaluated to the most relevant point(s) in clinicians’ workflow—for example, only when entering or signing an order, entering a diagnosis, administering a medication, or (most invasively) on every entry into the patient’s chart. Our stroke swallowing advisory was to trigger logic evaluation when the nurse prepares a medication for administration to a patient—specifically, at the time of barcode scanning the medication due (Figure 3).

#### Rule Logic

**Figure 4:**
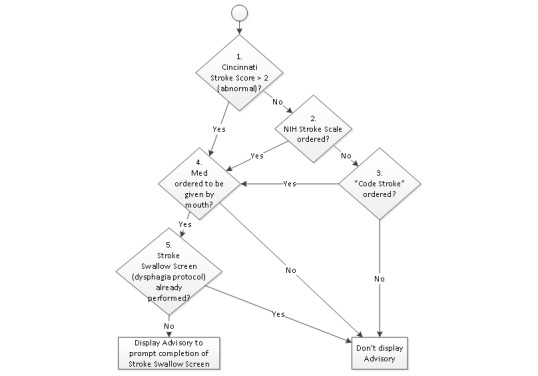
Decision tree for the advisory. NIH: National Institutes of Health.

**Figure 5:**
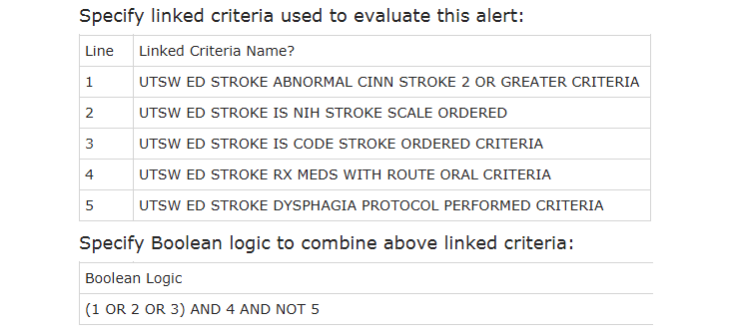
Screenshot of test specifying clinical decision support rule logic. CINN: Cincinnati; NIH: National Institutes of Health.

The rule logic for deciding whether a CDS advisory should appear to a clinician was first modeled as a decision tree (Figure 4), then specified as test tables (Figure 5). The specified logic checks for any of three potential indications that the patient has a known or suspected stroke diagnosis, then for a planned oral route of the barcode scanned medication, and finally whether the Stroke Swallow (dysphagia) Screen has been performed.

#### User Interface

**Figure 6:**
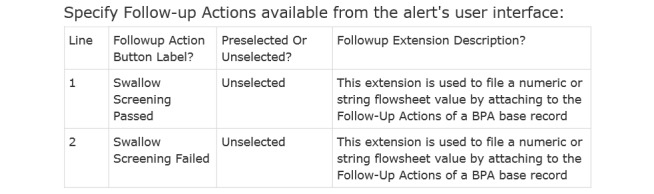
Screenshot of test specifying user interface actions for the advisory. BPA: Best Practice Advisory

In addition to specifying the wording on the advisory (not shown and which includes instructional diagrams and text for performing the Swallow Screen), acceptance tests can also specify what follow-up actions the clinician may be prompted to perform (Figure 6).

This requirement test specified that the nurse should be able to indicate directly from the alert’s UI whether the patient passed or failed the Swallow Screen, without having to leave the alert and navigate to the swallow screening flowsheet in another part of the chart. This follow-up action still populated the same flowsheet behind the scenes, however, for data consistency. Neither option was to be defaulted as pre-selected—both were specified to initially appear unselected.

#### System Actions

**Figure 7:**
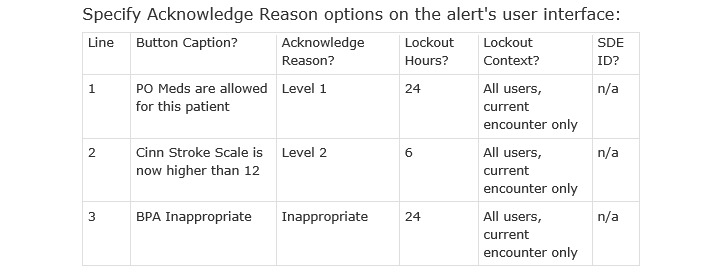
Screenshot of test specifying system actions following clinician response. BPA: Best Practice Advisory; PO: per os.

As an alternative to the prompted action, the clinician may select an “acknowledge reason” exception why the primary action was not taken, resulting in the system setting a “lock out” time to avoid repetitive firing, and optionally file a specific data element for data capture (Figure 7).

For instance, Line 1 of this test specifies that once a clinician determines oral medications are allowed for this patient, the alert should not fire on subsequent medication administrations during the current ED encounter for a lockout period of 24 hours, limiting alert fatigue.

### Test–Driven Development Cycle

#### Before Development

**Figure 8:**
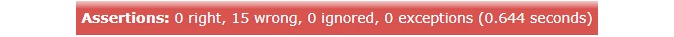
Screenshot of acceptance test: all assertions fail as expected prior to build.

Before development has begun, all test assertions should fail and do (Figure 8).

#### During Development

**Figure 9:**
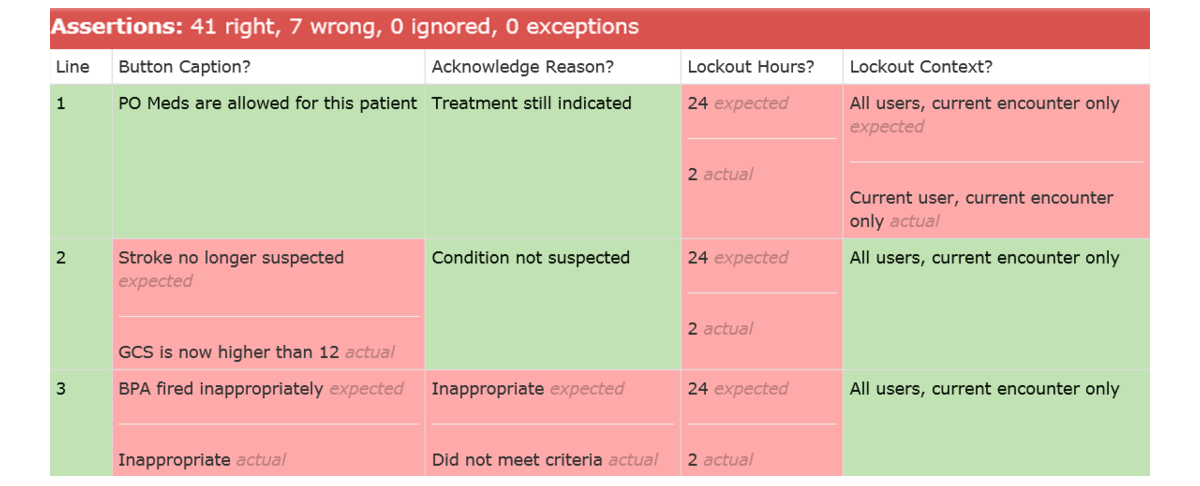
Screenshot of a test table included in the acceptance test suite: acceptance test partially passes following initial build. GCS: Glasgow Coma Scale; PO: per os.

During development, some tests begin to pass. When construction of the CDS advisory is complete, the test suite can indicate if any requirements are not yet met (Figure 9).

FitNesse automatically displays any discrepancies between expected and actual advisory design. On Line 1 in Figure 9, the Lockout Hours setting was specified as 24 hours but initially configured to 2 hours, which if unchanged would cause significant over-firing of the alert to busy nurses.

#### After Successful Development

**Figure 10:**
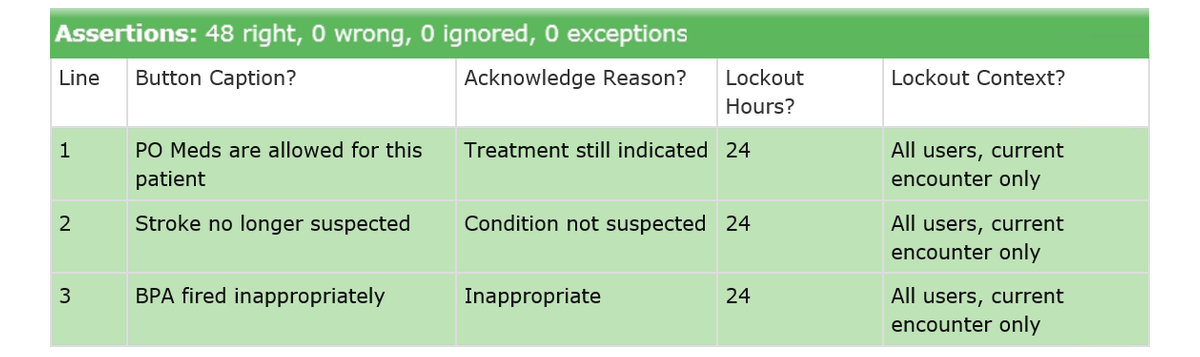
Screenshot of acceptance test assertions for "base" alert record, all passing following successful build. BPA: Best Practice Advisory; PO: per os.

Following completion of build and resolution of any discrepancies from specified requirements, the test page for the “base” alert record passes completely (Figure 10).

Similar test pages were developed to specify acceptance criteria for the 5 “criteria” records referenced by the base alert record (see Multimedia Appendices 1 and 2 ). The full test suite thus consisted of 6 test pages, encompassing 24 individual tests making 133 individual assertions. The total time to execute each test page and the full test suite are given in Table 2 (times are the average of 5 test suite executions). The full suite averages 0.933 seconds to run, most of which is suite set-up and wrap-up time. Each test page execution takes only 2-4 ms (0.002-0.004 s).

For reference, our current FitNesse regression test suite in production currently has 85 Test Pages, 6126 individual test Assertions, and runs in 165 seconds (2 min, 45 sec). Once the automated acceptance tests are fully passing, the CDS advisory then can be migrated from the Development environment to the Integrated Testing environment, and then to Production. The automated acceptance test suite is also added to the regression test suites in the latter two environments contemporaneously with migrating the CDS code, to ensure continued proper behavior in all environments.

**Table 2:** Test suite: number of tests and individual assertions, with execution times. NIH: National Institutes of Health.

Type	Test page name	Tests	Assertions	Time (s)
Base	Alert Stroke Suspected But No Swallow Screen	8	48	0.003
Criteria	Criteria Abnormal Cincinnati Stroke Scale	3	14	0.002
Criteria	Criteria NIH Stroke Scale Ordered	3	19	0.001
Criteria	Criteria Code Stroke Ordered	4	24	0.002
Criteria	Criteria Med With Oral Route	3	19	0.002
Criteria	Criteria Stroke Dysphagia Screen Performed	3	14	0.002
Suite	Suite Story Stroke Swallow Screen	24	138	0.869

### Iterative Development

#### Number of Iterations Required

Three 2-week development iterations were required for full implementation of this advisory, following requirements gathering with a user story and initial acceptance criteria.

- During a first 2-week iteration, automated acceptance tests were written and a first working version of the best practice advisory created and demonstrated. During testing, we discovered that the initial follow-up action specified by the test (a hyperlink to jump the nurse to the Swallow Screen documentation flowsheet) was not compatible with the trigger action desired (beginning medication administration).
- Accordingly, during a follow-on 2-week iteration, we pivoted to a different follow-up action to be taken from the advisory’s UI, which enabled the nurse to document the Stroke Swallow screen results directly from the advisory UI. This filed the nurse’s response to the identical Stroke Swallow documentation flowsheet row, while avoiding the need for the nurse to leave the advisory and jump to the flowsheet itself. Since the advisory’s UI also includes graphical instructions for performing the Stroke Swallow screen, this approach was well received by nursing representatives.
- During a third 2-week iteration, the alert was turned on in Production silently (not visible to end-users) to observe what situations triggered its firing. No over-firing in unwanted situations was detected. Under-firing was observed, due to frequent use in the ED of as-needed oral medication orders rather than scheduled medication orders. The criteria record’s rule determining whether oral meds were ordered originally used a property evaluating for oral scheduled medications. This rule was re-specified to include an additional property evaluating for as-needed oral medications as well. After development to pass the revised test, the modified rule was re-migrated to Production.

#### Go-Live in Production

The alert was re-observed silently in Production for approximately 24 hours prior to enabling its display to end-users. Investigation of the alert’s criteria evaluation for both real ED patients and test patients confirmed that the alert was behaving as expected in Production. Following “go-live” of the visible alert, no customer-logged “tickets” for aberrant alert behavior (eg, firing in unintended locations or situations) were received.

## Discussion

### Principal Results

Defects and unintended consequences occur too commonly in CDS advisories present in modern complex EHRs. Test–driven development offers one approach to help achieve higher reliability. In this study, we used open source software (FitNesse) to create “executable requirements” covering multiple important structural and behavioral dimensions of CDS advisory design: restrictions to applicable clinical settings, trigger(s) to invoke rule evaluation, rule logic, UI design, and system responses to clinician selections. This work demonstrates that acceptance TDD can feasibly be applied to configuring CDS advisories in a commercial EHR, generating suites of automated acceptance and regression tests.

### Comparison With Prior Work

User-centered design methods now being applied in health care seek to optimize clinician and patient experience with software and include the equivalent of iterative manual acceptance testing [24-27]. We consider the use of automated acceptance and regression testing complementary, and a means of capturing insights from user-centered design in explicitly testable ways to ensure accurate implementation. Sophisticated automated generation of test cases for complex CDS tool logic has been previously described, to identify and test all possible guideline-permitted decision paths [28,29]. In those studies, clinician and patient user acceptance testing of interactions with the CDS tool itself remained manual, though testing of the CDS logic was fully automated.

### Limitations

In this study, we demonstrated the feasibility of using TDD for CDS configuration in a commercial EHR: investigation over a longer period of adoption will be needed to measure the effect of TDD on CDS tools’ quality in production.

The example chosen shows application of TDD to only one type of CDS (best practice advisories), in an advisory executing simple logic. However, the FitNesse framework in our experience can be readily applied to specifying more complex CDS rule logic assessing a wide variety of patient-specific data in the EHR and to testing many other aspects of EHR and non-EHR system configuration. For instance, we have applied FitNesse automated testing to:

- ensuring conformance with data business rules not enforced directly in software (eg, “If a provider is marked as participating in the EHR Incentive Program, they should also have their e-Prescribing flag set to Yes”)
- specifying expected contents of tables with potential for major downstream impact if unexpectedly changed (eg, exact contents of the Provider Type and Encounter Type look-up tables, used extensively in CDS targeting, in reporting, and in a variety of operational uses)
- cross-system testing of mutually consistent configuration (eg, for the exact operating room location of vital sign monitoring equipment used by anesthesiologists, validate 100% consistency between middleware software and the EHR, to ensure vital signs are always interfaced to the correct surgical patient’s record)

Given this versatility, we expect automated acceptance TDD to prove readily applicable to other types of CDS (such as order sets, cascading order questions, and rule-driven banners).

Another potential limitation is that FitNesse by design tests software “under the hood”; that is, under the UI level. FitNesse purposefully tests the business logic and data storage layers driving important application behavior, ideally insulated by modular design from minor modifications to the UI. Automated testing through the UI generally requires more maintenance and is more time-consuming and expensive to configure [13]. Nonetheless, testing through the UI can be necessary in some circumstances, for instance if the EHR software embeds certain business logic completely within the UI layer (without reference to business logic modules or configuration tables). To test those aspects, FitNesse would need to be augmented with an automated testing tool operating through the UI. We use such a tool (ie, TestComplete, SmartBear Software) for automated “journey” or scenario testing by a simulated user, complementary to automated TDD and regression testing of EHR configuration using FitNesse.

### Conclusions

Automated acceptance testing and continuous regression testing of CDS configuration in a commercial EHR proves feasible with open source software. The problem of EHR safety is multifaceted, and multiple safety-enhancing approaches will almost certainly be needed [30]. Automated TDD offers one potential contribution towards achieving high-reliability EHR systems.

As another benefit, clinician frustration with the EHR can be reduced by judiciously limiting interruptive alerts to truly relevant circumstances where pop-up advice is seen as helpful, not extraneous [31]. Vetting acceptance tests with clinicians elicits their input on crucial configuration details early during initial CDS design, as well as iteratively during rapid-cycle evolutionary development.

## Appendix

Multimedia Appendix 1 FitNesse test tables for Stroke Swallow Advisory - main alert record.

Multimedia Appendix 2 FitNesse test tables for Stroke Swallow Advisory - criteria records.

## Abbreviations

BPA: Best Practice Advisory
CDS: clinical decision support
EHR: electronic health record
GCS: Glasgow Coma Scale
IT: information technology
NIH: National Institutes of Health
PO: per os
SQL: structured query language
TDD: test–driven development
